# Kappa-carrageenan-Functionalization of octacalcium phosphate-coated titanium Discs enhances pre-osteoblast behavior and osteogenic differentiation

**DOI:** 10.3389/fbioe.2022.1011853

**Published:** 2022-10-20

**Authors:** Wei Cao, Jianfeng Jin, Gang Wu, Nathalie Bravenboer, Marco N. Helder, Engelbert A. J. M. Schulten, Rommel G. Bacabac, Janak L. Pathak, Jenneke Klein-Nulend

**Affiliations:** ^1^ Department of Oral Cell Biology, Academic Centre for Dentistry Amsterdam (ACTA), University of Amsterdam and Vrije Universiteit Amsterdam, Amsterdam Movement Sciences, Amsterdam, Netherlands; ^2^ Department of Oral and Maxillofacial Surgery/Oral Pathology, Amsterdam University Medical Centers and Academic Centre for Dentistry Amsterdam (ACTA), Vrije Universiteit Amsterdam, Amsterdam Movement Sciences, Amsterdam, Netherlands; ^3^ Department of Clinical Chemistry, Amsterdam University Medical Centers, Vrije Universiteit Amsterdam, Amsterdam Movement Sciences, Amsterdam, Netherlands; ^4^ Department of Physics, Medical Biophysics Group, University of San Carlos, Cebu City, Phlilippines; ^5^ Guangzhou Key Laboratory of Basic and Applied Research of Oral Regenerative Medicine, Affiliated Stomatology Hospital of Guangzhou Medical University, Guangdong Engineering Research Center of Oral Restoration and Reconstruction, Guangzhou, China

**Keywords:** bone regeneration, κ-carrageenan, implant, OCP-coating, osteogenic differentiation, osteointegration, pre-osteoblast, titanium

## Abstract

Bioactive coatings are promising for improving osseointegration and the long-term success of titanium dental or orthopaedic implants. Biomimetic octacalcium phosphate (OCP) coating can be used as a carrier for osteoinductive agents. κ-Carrageenan, a highly hydrophilic and biocompatible seaweed-derived sulfated-polysaccharide, promotes pre-osteoblast activity required for bone regeneration. Whether κ-carrageenan can functionalize OCP-coating to enhance osseointegration of titanium implants is unclear. This study aimed to analyze carrageenan-functionalized biomimetic OCP-coated titanium structure, and effects of carrageenan functionalization on pre-osteoblast behavior and osteogenic differentiation. Titanium discs were coated with OCP/κ-carrageenan at 0.125–2 mg/ml OCP solution, and physicochemical and biological properties were investigated. κ-Carrageenan (2 mg/ml) in the OCP coating of titanium discs decreased the pore size in the sheet-like OCP crystal by 41.32%. None of the κ-carrageenan concentrations tested in the OCP-coating did affect hydrophilicity. However, κ-carrageenan (2 mg/ml) increased (1.26-fold) MC3T3-E1 pre-osteoblast spreading at 1 h i.e., κ-Carrageenan in the OCP-coating increased pre-osteoblast proliferation (max. 1.92-fold at 2 mg/ml, day 1), metabolic activity (max. 1.50-fold at 2 mg/ml, day 3), and alkaline phosphatase protein (max. 4.21-fold at 2 mg/ml, day 3), as well as matrix mineralization (max. 5.45-fold at 2 mg/ml, day 21). κ-Carrageenan (2 mg/ml) in the OCP-coating increased gene expression of *Mepe* (4.93-fold) at day 14, and *Runx2* (2.94-fold), *Opn* (3.59-fold), *Fgf2* (3.47-fold), *Ocn* (3.88-fold), and *Dmp1* (4.59-fold) at day 21 in pre-osteoblasts. In conclusion, κ-carrageenan modified the morphology and microstructure of OCP-coating on titanium discs, and enhanced pre-osteoblast metabolic activity, proliferation, and osteogenic differentiation. This suggests that κ-carrageenan-functionalized OCP coating may be promising for *in vivo* improvement of titanium implant osseointegration.

## 1 Introduction

Titanium implants are widely used in dentistry, as well as for critical-sized bone defect reconstruction in orthopaedics (Haugen and Chen, 2022). Dental or orthopaedic implant osseointegration is crucial for prolonged implant success (Haugen and Chen, 2022). Osseointegration, the direct structural and functional connection between ordered living bone and the surface of a load-carrying implant, involves the incorporation of nonvital components of the living bone leading to an efficient, reliable, and predictable anchorage mechanism (Brånemark et al., 2001). However, titanium implants lack the biological properties needed for osseointegration, such as osteoconductivity and osteoinductivity (Das et al., 2019). Compromised osseointegration might lead to implant failure, which occurs more often in systemic and local diseases, such as diabetes, periodontitis, and osteoporosis, where the inflammatory environment alters osteoblast and osteoclast function thereby impairing the bone regeneration process (Chen et al., 2013; Haugen and Chen, 2022). Titanium implant surface modification approaches ensuring implant success in patients with inflammatory diseases are still in high demand (Das et al., 2019). Moreover, cost-effective approaches to improve titanium implant surface modifications, that can be easily translated to the clinic, need to be further explored. Various physicochemical and biological strategies are already available to improve the implant’s biological properties, including calcium phosphate (CaP) coating (Liu et al., 2007; Kim et al., 2017), sandblast and acid etching (Wang et al., 2020), doping silicon and copper ions into high-energy shot peening-assisted micro-arc oxidation-treated coatings (Shen et al., 2020), and plasma spray (Wang et al., 2022). Among these methods, CaP coating is the most efficient and least expensive (Kim et al., 2017). CaP coatings are hydrophilic, which improves cell attachment, proliferation, and differentiation, leading to enhanced osseointegration and success rate of dental implants (Zheng et al., 2014; Kim et al., 2017). CaP-coated implants show good biological activity and osteoconductivity, but osteoinductivity is poor (Barrère et al., 1999). To tackle this issue, the incorporation of biologically active agents, e.g. bone morphogenetic protein-2 (BMP2), in a CaP-coating on an implant surface may be used to improve implant osseointegration.

Different strategies have been developed to lay down CaP-coatings on titanium implants, such as electrostatic or plasma spraying (Siebers et al., 2004), coating *via* sol gel phase shifting (Shen et al., 2015), high-temperature sintering (Kim et al., 2017), and magnetron sputtering (Wang et al., 2020). High-temperature sintering and electrostatic approaches destroy implant biological activity (Kim et al., 2017). A biomimetic CaP coating guarantees a slow, gradual, and local release of bioactive agents (e.g., BMP2) thereby mimicking the natural release of these factors from the bone matrix under physiological conditions (Liu et al., 2018). Also, biomimetic CaP coatings are promising as drug carrier. A CaP coating on scaffolds can be achieved by first precipitating amorphous CaP (ACP), followed by octacalcium phosphate (OCP) deposition including a drug or bioactive agent of interest (Wu et al., 2010).

OCP (Ca_8_(HPO_4_)_2_(PO_4_)_4_·5H_2_O) is one of the precursors of hydroxyapatite during bone mineralization. OCP crystals are deposited on titanium through a two-step procedure (Barrère et al., 2001). A CaP coating on scaffolds is achieved by first precipitating amorphous CaP (ACP), followed by octa-CaP deposition, including a drug or bioactive agent of interest. A prominent advantage of using such an osteogenic agent/drug-containing CaP coating is that the biological activity of the agent/drug is preserved during coprecipitation with inorganic components at physiological temperature (37°C) and pH (7.4) (Lin et al., 2020). In addition, the crystal structure of CaP formed is more akin to that of the bone mineral than of tri-/tetracalcium phosphate and hydroxyapatite, which are produced at exceedingly high temperatures (>1,000°C) (Kokubo et al., 1990). Moreover, a minimum dose of drugs or biological agents can be incorporated in CaP coating to provide sustained drug or biological agent release for weeks in a cell-mediated manner (Wu et al., 2010). BMP2 and polymers have been incorporated in CaP coating to improve the biological activity and osteoinductivity of CaP-coated implants (Fini et al., 2002; Lin et al., 2020). CaP coating with BMP2 improves implant osteoinductivity (Vehof et al., 2001). Unfortunately, the clinical use of BMP2 is very expensive (Zheng et al., 2014). Therefore, the search for alternative cost-effective bioactive agents that can be incorporated in biomimetic CaP coating on an implant surface to improve implant osseointegration is still ongoing.

κ-Carrageenan is a natural polysaccharide extracted from diverse red seaweeds with abundant sulfate groups (Du et al., 2016). κ-Carrageenan has been used widely in the food industry to gel, thicken, and stabilize food products for decades, and its safety has been recognized based on a large database of animal studies (Geonzon et al., 2019). The structure of κ-carrageenan is similar to glycosaminoglycan (GAG), which is naturally found in human bone and cartilage. κ-Carrageenan has both chondrogenic and osteogenic potential based on its chondroitin-4-sulfate and dermatan sulfate (Mokhtari et al., 2019). Therefore, κ-carrageenan is considered to be a potent factor that can induce bone regeneration. Importantly, κ-carrageenan-functionalized graphene oxide (GO) has been shown to enhance the adhesion and proliferation of MC3T3-E1 pre-osteoblasts (Liu et al., 2014). Carrageenan nanocomposite hydrogel incorporated with dimethyloxallylglycine and whitlockite nanoparticles enhances osteogenic gene expression, e.g., RUNX2, COL1α-1, and OPN (Mokhtari et al., 2019). Collagen-hydroxyapatite/κ-carrageenan (COL-HAP/κ-Car) composite has been shown to have similar structural characteristics with natural bone (Feng et al., 2017). Moreover, κ-carrageenan/silk fibroin bioactive composite scaffolds are biocompatible, and can induce precursor cell proliferation and osteogenic differentiation (Nourmohammadi et al., 2017). An ideal therapeutic agent for bone substitute functionalization should have the potential to induce cellular activities required for bone regeneration including cell migration, adhesion, spreading, proliferation, and osteogenic differentiation (Zamani et al., 2018). Earlier we found that κ-carrageenan promotes migration, adhesion, spreading, and osteogenic differentiation of MC3T3-E1 pre-osteoblasts *in vitro* (Cao et al., 2021). Therefore, κ-carrageenan could be a potent factor to improve implant osteoinductivity and osseointegration when incorporated in a CaP coating of titanium implants.

In the present study, we aimed to functionalize titanium implants by incorporating κ-carrageenan at 0.125, 0.25, 0.5, 1, and 2 mg/ml in OCP coating by depositing κ-carrageenan and OCP simultaneously layer-by-layer on a titanium surface using a biomimetic co-precipitation technique. The physicochemical properties of the OCP coating without/with κ-carrageenan were characterized. MC3T3-E1 pre-osteoblast adhesion, metabolic activity, morphology, spreading, proliferation, alkaline phosphatase (ALP) activity, matrix mineralization, and osteogenic gene expression were assessed after seeding on OCP-coated titanium discs without/with κ-carrageenan.

## 2 Materials and methods

### 2.1 κ-Carrageenan

κ-Carrageenan was kindly supplied by Tokyo Chemical Industry Co., Ltd. (Tokyo, Japan), and was prepared as described earlier (Du et al., 2016). The freeze-dried pure κ-carrageenan (100%, wt/vol) was dissolved in deionized water at 28 mg/ml (stock solution) under stirring overnight at room temperature, sterilized by heating for 2 min to 90°C, and stored at 4°C.

### 2.2 Octacalcium phosphate coating without/with κ-carrageenan

Titanium discs (diameter: 10 mm; height: 1 mm; Baozhong, Shanxi, China) were immersed for 24 h at 37°C in 50 ml of a solution (adjusted to pH 6.5) containing 684 mM NaCl, 12.5 mM CaCl_2_⋅2H_2_O, 5 mM Na_2_HPO_4_⋅2H_2_O, 21 mM NaHCO_3_, and 7.5 mM MgCl_2_⋅6H_2_O. An amorphous CaP layer was achieved in the presence of 7.5 mM MgCl_2_⋅6H_2_O to inhibit crystal growth. At the end of 24 h incubation, the pH of the solution had increased from 6.5 to 8.0. Coated discs were cleaned ultrasonically in deionized water for 10 min, and air-dried overnight at ambient temperature. The thin, dense layer of amorphous CaP formed served as a seeding substratum for the deposition of another more crystalline OCP layer. OCP-coated discs without/with κ-carrageenan were prepared by immersion of the amorphous CaP-coated discs for 48 h at 37°C in 50 ml of a super-saturated solution of OCP containing 40 mM HCl, 4 mM CaCl_2_⋅2H_2_O, 136 mM NaCl, and 2 mM Na_2_HPO_4_⋅2H_2_O (pH 7.4), as well as 0, 0.125, 0.25, 0.5, 1, or 2 mg κ-carrageenan/ml. The concentration of κ-carrageenan in food additives is 1–3 mg/ml (Du et al., 2016). Previously we showed that κ-carrageenan (0.125–2 mg/ml) dose-dependently increases pre-osteoblast proliferation and metabolic activity, with a maximum effect at 2 mg/ml (Cao et al., 2021). κ-Carrageenan (0.5 and 2 mg/ml) increases osteogenic differentiation by enhancing alkaline phosphatase activity, matrix mineralization, and expression of osteogenic markers *Opn*, *Dmp1*, and *Mepe* (Cao et al., 2021). All chemical components in the supersaturated OCP solution used for preparing the OCP-coated titanium discs should be completely dissolved to allow the development of the CaP coating. However, κ-carrageenan at >3 mg/ml resulted in OCP precipitation in the solution (Supplementary Figure S2). Therefore we used κ-carrageenan at 2 mg/ml OCP solution as the highest concentration (Supplementary Figure S2). Titanium discs with OCP coatings without/with κ-carrageenan were air-dried overnight at ambient temperature and used for experiments.

### 2.3 Characterization of octacalcium phosphate coating without/with κ-carrageenan

#### 2.3.1 Surface morphology

The surface morphology of OCP-coated discs without/with κ-carrageenan was observed by scanning electron microscopy (SEM; S-3400N II, Hitachi, Tokyo, Japan). Discs were coated with a layer of gold using magnetron sputter (Shinkku VD, MSP-1S, Mito, Japan), and imaged using an SEM with an accelerating voltage of 15 kV.

#### 2.3.2 Elemental composition

The elemental composition of OCP coatings without/with κ-carrageenan was evaluated with energy dispersive X-ray spectroscopy (EDX; Model 55i, IXRF, Austin, Texas, United States) analysis. Discs were assayed in triplicate.

#### 2.3.3 Surface chemical composition and crystalline phase

Raman spectroscopy (DXR3, Thermo Fisher Scientific, Waltham, MA, United States) was used to identify functional groups, chemical interactions, and possible alteration of OCP coatings with κ-carrageenan. To identify the crystalline phases of OCP coatings without/with κ-carrageenan, X-ray diffraction (XRD) analysis was performed using an X-ray diffractometer (Empyrean, Malvern, United Kingdom). Discs were assayed in triplicate.

#### 2.3.4 Hydrophilicity

To determine the hydrophilicity of OCP coatings without/with κ-carrageenan, the static water contact angle was measured. A video contact angle system (Sony, Tokyo, Japan) was used to capture water contact angle images. The water contact angle was determined *via* ImageJ. Discs were analyzed in triplicate.

### 2.4 Protein release from octacalcium phosphate coating without/with κ-carrageenan

Protein release from OCP-coated discs without/with κ-carrageenan was determined using fluorescein isothiocyanate (FITC)-bovine serum albumin (BSA). Discs were incubated for 24 h in 1 ml PBS containing 10 mg FITC-BSA for up to 7 days at 37°C. Hundred μl samples of the solution were taken at 1, 2, 12, and 24 h, as well as after 7 days. Thousand μl PBS was replaced with fresh PBS after taking each sample. Fluorescent images of the discs were made using a fluorescent microscope (Leica, Wetzlar, Germany), and fluorescence intensity was quantified using ImageJ software (https://imagej.net/Fiji.Downloads) (Jin et al., 2019). Serial dilutions of FITC-BSA in PBS were used as a standard curve. The fluorescence intensity was monitored at 485 nm (excitation) and 528 nm (emission) in a microplate reader (Synergy, BioTek™, Winooski, VT, United States).

Protein adsorption onto titanium discs with or without κ-carrageenan was performed in α-MEM containing 10% fetal calf serum (FCS, Gibco, Paisly, United Kingdom). After incubation for 1, 12, or 24 h at 37°C, the discs were transferred into a new 48-well plate (one disc per well), and washed thoroughly with PBS. Five-hundred µl 1% sodium dodecyl sulfate (SDS; Sigma, St. Louis, MO, United States) was added per well for quantification of protein adsorption. The amount of protein was determined using a BCA Protein Assay Reagent kit (Thermo Fisher Scientific, Rockford, IL, United States), and the absorbance was read at 540 nm with a microplate reader (Synergy, BioTek™, Winooski, VT, United States). (Supplementary Figure S3)

### 2.5 MC3T3-E1 pre-osteoblast culture and bioactivity

#### 2.5.1 Cell culture and seeding onto octacalcium phosphate-coated discs without/with κ-carrageenan

MC3T3-E1 pre-osteoblast (American Type Culture Collection, Manassas, VA, United States) were grown and maintained in α-Minimum Essential Medium (α-MEM; Gibco, Paisly, United Kingdom), supplemented with 10% fetal bovine serum (FBS; Gibco), and 1% PSF (antibiotic antimycotic solution, Sigma-Aldrich^®^, St. Louis, MO, United States), in a humidified incubator with 5% CO_2_ in air at 37°C. After reaching 85% confluency, cells were detached using 0.25% trypsin (Gibco) and 0.1% ethylenediaminetetraacetic acid (EDTA, Merck, Darmstadt, Germany) in phosphate buffered saline (PBS; Gibco) at 37°C. Cells were then resuspended in α-MEM with 10% FBS and antibiotics, seeded in 96, 48, or 24-well culture plates (Greiner, Bio-one, Alphen a/d Rijn, Netherlands), and cultured for different periods, from 1 h up to 21 days, dependent on the outcome parameter measured (see below).

Cell seeding was done by seeding MC3T3-E1 pre-osteoblasts (5 × 10^3^ cells/disc (cell proliferation), 1×10^4^ cells/well (cell spreading, paxillin immunofluorescence staining), 1×10^5^ cells/disc (cell metabolic activity, alkaline phosphatase (ALP) activity and protein assay, alizarin red staining, and osteogenic gene expression), onto OCP-coated titanium discs without/with κ-carrageenan, which were put in 48-well culture plates (Greiner). Cell-seeded discs were incubated for 8 h in 5% CO_2_ in air at 37°C to allow cell attachment. Then osteogenic medium (250 µl/well) was added, and cell-seeded discs were cultured for up to 21 days. The culture medium was changed every 2 days. Cell adhesion, metabolic activity, morphology, spreading, proliferation, ALP activity, matrix mineralization, and osteogenic gene expression were assessed as described below. Ascorbic acid (Sigma; 0.1 mg/ml) and β-glycerophosphate (phosphate donor; Sigma; 10 mM) were added to the culture medium for determination of osteogenic gene expression, ALP activity, ALP protein, and matrix mineralization.

#### 2.5.2 Cell adhesion and spreading

MC3T3-E1 pre-osteoblasts were seeded at 1×10^4^ cells/well onto OCP-coated titanium discs without/with κ-carrageenan (2 mg/ml). Discs were put into 48-well plates (Greiner; 1 disc/well) and incubated for 1 h. After incubation, the medium was removed, discs were washed twice with PBS, and fixed in 2.5% glutaraldehyde overnight at 4°C. After washing twice with deionized water, the discs were dehydrated using graded ethanol series (50, 70, 80, 90, 100%). Then, discs were air-dried overnight with hexamethyldisilane (HMDS; Sigma) in a chemical hood, followed by coating with a layer of gold using a magnetron sputter. Discs were imaged using an SEM with an accelerating voltage of 15 kV. For determination of cell surface area, cell length, and cell width, three regions of interest (ROI), measuring 250 × 175 µm, were defined on SEM images of OCP-coated titanium discs with/without κ-carrageenan. One ROI was positioned in the center of each disc, and two ROIs were evenly spaced (200 µm) from the center of each disc in opposite directions. The total number of cells in the three ROIs ranged from 20 to 25. Cell surface area, cell length, and cell width were calculated using an Analyze Particles cell plugin in ImageJ software.

#### 2.5.3 Paxillin immunofluorescence staining

One hour after seeding MC3T3-E1 pre-osteoblasts onto OCP-coated titanium discs without/with κ-carrageenan (2 mg/ml), cells were fixed with 4% paraformaldehyde solution for 15 min at 37°C, treated with 0.2% Triton X-100 (Sigma) for 10 min, and non-essentially bound substances were blocked in 5% BSA for 30 min. Paxillin expression was analyzed by immunofluorescence staining using rhodamine-phalloidin cytoskeleton dye (Invitrogen, Fisher Scientific, Carlsbad, CA, United States) and p-paxillin pTy31 polyclonal rabbit IgG (ab32084, Abcam, Cambridgeshire, United Kingdom). The secondary antibody used was Alexa Fluor-488 goat anti-rat IgG (Abcam). Nuclei were stained blue with 1 μg/ml DAPI (Sigma). After glycerol mounting, cell imaging was performed by laser scanning confocal microscopy (LSCM; Nikon, A1R/A1, Tokyo, Japan). Fluorescence microscopy was also used to visualize paxillin at 488 nm wavelength, and ImageJ software was used for paxillin area quantification. For paxillin area quantification, three ROIs, measuring 50 × 50 µm, of each OCP-coated titanium disc with/without κ-carrageenan, were defined on fluorescent images. One ROI was positioned in the center of each disc, and two ROIs were evenly spaced (200 µm) from the center of each disc in opposite directions. Paxillin was visualized using fluorescence microscopy at 488 nm wavelength. ImageJ software was used for paxillin area quantification. The total cell number in the three ROIs ranged from 20 to 25.

#### 2.5.4 Cell proliferation

MC3T3-E1 pre-osteoblast proliferation was assessed by DNA content quantification. Cells were cultured onto OCP-coated titanium discs without/with κ-carrageenan at 5 × 10^3^ cells/disc in 48-well plates (Greiner) up to 3 days. Cell lysate was collected using lysis buffer, and DNA content per well was determined with the Cyquant Cell Proliferation Assay (Molecular Probes, Eugene, OR, United States) according to the manufacturer’s protocol. Fluorescence intensity was read at 485 nm (excitation) and 528 nm (emission) in a microplate reader (Synergy).

#### 2.5.5 Cell metabolic activity

To assess cell metabolic activity, MC3T3-E1 pre-osteoblasts were seeded on OCP-coated titanium discs without/with κ-carrageenan at 1×10^5^ cells/disc in 48-well plates (Greiner), and cultured up to 7 days. The medium was removed, cells were washed with PBS, and α-MEM with 10% FBS and antibiotics was added. The PrestoBlue™ Assay (Invitrogen) was used to evaluate cell metabolic activity according to the manufacturer’s instructions. In short, PrestoBlue™ reagent was added to the cells (10%, vol/vol), followed by 30 min incubation in a 5% CO_2_ in air incubator with a humidified atmosphere at 37°C. The medium was harvested (100 μl/well) and transferred into a 96-wells black microplate (Greiner). Fluorescence intensity was determined at a wavelength of 560 nm (excitation) and 590 nm (emission), and quantified using a Multiskan™ FC Microplate Photometer (Thermo Fisher Scientific). Prestoblue™ fluorescence was linearly associated with DNA content (data not shown).

#### 2.5.6 Alkaline phosphatase protein, alkaline phosphatase activity, and total protein assay

To assess the osteogenic phenotype of MC3T3-E1 pre-osteoblasts, cells were seeded at 1×10^5^ cells/disc coated with OCP without or with κ-carrageenan and cultured for 3 or 7 days on OCP-coated titanium discs without/with κ-carrageenan, and ALP activity and protein content determined. ALP protein on OCP-coated titanium discs without/with κ-carrageenan was stained at 3 and 7 days. The culture medium was removed and discs were washed 2 times with PBS, and fixed with 4% formaldehyde in PBS for 15 min at 37°C. ALP protein staining kit (Beyotime, Shanghai, China) was used for the colorimetric detection of ALP intensity, using 30 min incubation at 37°C. Optical images were taken using a stereomicroscope. Quantification of ALP protein was performed using ImageJ software. Note that the images were converted to gray scale 8. The stained ALP protein area was determined using the plugin of Analyze Particles. Three independent experiments providing 9 images of titanium discs (*n* = 3) were performed. To determine ALP activity, p-nitrophenyl phosphate (Merck) at pH 10.3 was used as a substrate. The absorbance was read at 405 nm in a microplate reader (Synergy). The amount of total protein was determined using a BCA Protein Assay Reagent kit (Thermo Fisher Scientific), and the absorbance was read at 540 nm with a microplate reader (Synergy). ALP activity was expressed as nmol/μg total protein.

#### 2.5.7 Alizarin red staining and mineralized nodule quantification

Matrix mineralization was analyzed by alizarin red staining of MC3T3-E1 pre-osteoblasts seeded at 1×10^5^ cells/disc coated with OCP without or with κ-carrageenan (0.5 or 2 mg/ml) and cultured for 21 days. Cells were fixed with 4% paraformaldehyde for 15 min, followed by rinsing with deionized water. Two hundred μl of 2% alizarin red solution in water, pH 4.3 (Alizarin Red S, Sigma-Aldrich, Los Angeles, CA, United States), was added per well for 30 min at room temperature. Then cells were washed with deionized water, and mineralization was quantified by dissolving the (red) mineralized matrix in 10% (vol/vol) cetylpyridinium chloride (Sigma) in 10 mM sodium phosphate solution (Sigma). Discs were de-stained for 1 h in 200 μl cetylpyridinium chloride solution on a rocking table, and the absorbance was read at 620 nm with a Multiskan FC (Thermo Fisher Scientific).

#### 2.5.8 Osteogenic gene expression

Total RNA was isolated from the MC3T3-E1 pre-osteoblasts seeded at 1 × 10^5^ cells/disc coated with OCP without/with κ-carrageenan after 1, 14, and 21 days of culture using an Invitrogen RNA isolation kit (Invitrogen). cDNA synthesis was performed using 0.5–1 μg total RNA in 20 μl reaction mix consisting of 5 units Transcriptor Reverse Transcriptases (Roche Diagnostics, Basel, Switzerland), 1 mM of each dNTP (Invitrogen), 0.08 A_260_ units random primers (Roche Diagnostics), and 1x concentrated Transcriptor Reverse Transcriptase reaction buffer (Roche Diagnostics). Real-time PCR (RT-PCR) reactions were performed using the LightCycler^®^ 480 SYBR green I Master reaction mix according to the manufacturer’s instructions (Roche Diagnostics) in a Light Cycler 480 (Roche Diagnostics), and relative housekeeping gene expression (PBGD) and relative target gene expression, i.e. runt-related transcription factor 2 (*Runx2*), osteocalcin (*Ocn*), fibroblast growth factor 2 (*Fgf2*), dentin matrix protein 1 (*Dmp1*), and osteopontin (*Opn*) were determined. Primers (Invitrogen) used for RT-PCR are listed in Table 1. Values of target gene expression were normalized for *PBGD* gene expression.

**TABLE 1:** Primer sequences used for real-time PCR.

Target gene	Primer sequence	
*Runx2*	Forward	ATGCTTCATTCGCCTCAC
	Reverse	ACT​GCT​TGC​AGC​CTT​AAA​T
*Opn*	Forward	AGT​GAT​GAA​AGA​TGG​GCA​ACT
	Reverse	TCT​GGA​CCA​TCT​TCT​TGC​TGA
*Fgf2*	Forward	GGC​TTC​TTC​CTG​CGC​ATC​CA
	Reverse	TCC​GTG​ACC​GGT​AAG​TAT​TG
*Ocn*	Forward	CAG​ACA​CCA​TGA​GGA​CCA​TCT​T
	Reverse	GGT​CTG​ATA​GCT​CGT​CAC​AA
*Mepe*	Forward	GGA​GCA​CTC​ACT​ACC​TGA​C
	Reverse	TAGGCACTGCCACCATGT
*Dmp1*	Forward	CGG​CTG​GTG​GAC​TCT​CTA​AG
	Reverse	CGG​GGT​CGT​CGC​TCT​GCA​TC
*PBGD*	Forward	AGT​GAT​GAA​AGA​TGG​GCA​ACT
	Reverse	TCT​GGA​CCA​TCT​TCT​TGC​TGA

Runx2, Runt-related transcription factor-2; Opn, osteopontin; Fgf2, fibroblast growth factor-2; Ocn, osteocalcin; Mepe, matrix extracellular phosphoprotein; Dmp1, dentin matrix protein-1; PBGD, porphobilinogen deaminase.

### 2.6 Statistical analysis

Data are presented as mean ± standard deviation (SD). Data were analyzed using Graphpad Prism^®^ 7.0 (GraphPad Software Inc., La Jolla, CA, United States). One-way analysis of variance (ANOVA) with Bonferroni’s post hoc test was used to test differences between groups. A *p*-value <0.05 was considered statistically significant.

## 3 Results

### 3.1 Surface morphology and elemental composition of octacalcium phosphate-coated titanium discs without/with κ-carrageenan

**FIGURE 1:**
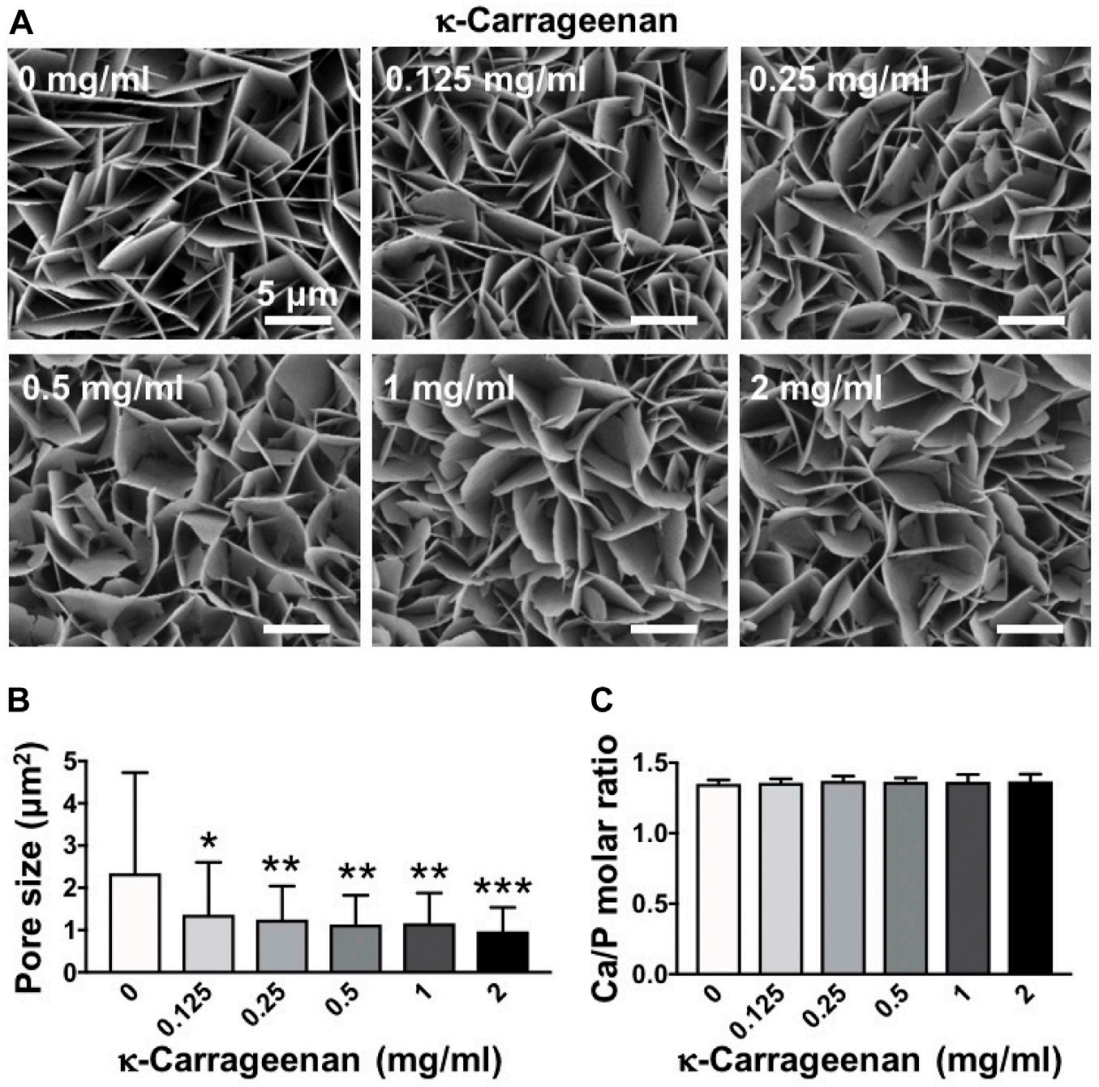
Dose-dependent effect of κ-carrageenan in octacalcium phosphate (OCP)-coating of titanium discs on surface morphology. **(A)** SEM micrographs. **(B)** Pore size. **(C)** Calcium/phosphate ratio. Values are mean ± SD. *n* = 9 from 3 independent experiments. Significant effect of κ-carrageenan, *p < 0.05, **p < 0.01, ***p < 0.001. Bar: 5 μm.

SEM images were taken to show the effect of increasing κ-carrageenan concentration (0–2 mg/ml) in OCP-coatings on surface morphology and microstructure. κ-carrageenan did affect the morphology, by changing the more straight sheet-like structure (0 mg/ml) into a more curved sheet-like structure at all concentrations tested (Figure 1A). κ-carrageenan significantly reduced the pore size in the sheet-like structure of OCP crystal by 41.32% (Figure 1B). The elemental composition of OCP-coated titanium discs without/with κ-carrageenan surface functionalization was analyzed by EDX spectroscopy. EDX spectra indicated the presence of calcium (Ca; 36.73 ± 1.44 wt%), and phosphate (P; 20.32 ± 1.08 wt%). To assess whether κ-carrageenan affects OCP crystallinity, the Ca/P ratio was calculated (Figure 1C). κ-carrageenan did not affect the Ca/P ratio of the OCP coating.

### 3.2 Chemical composition and hydrophilicity of octacalcium phosphate-coated titanium discs without/with κ-carrageenan

**FIGURE 2:**
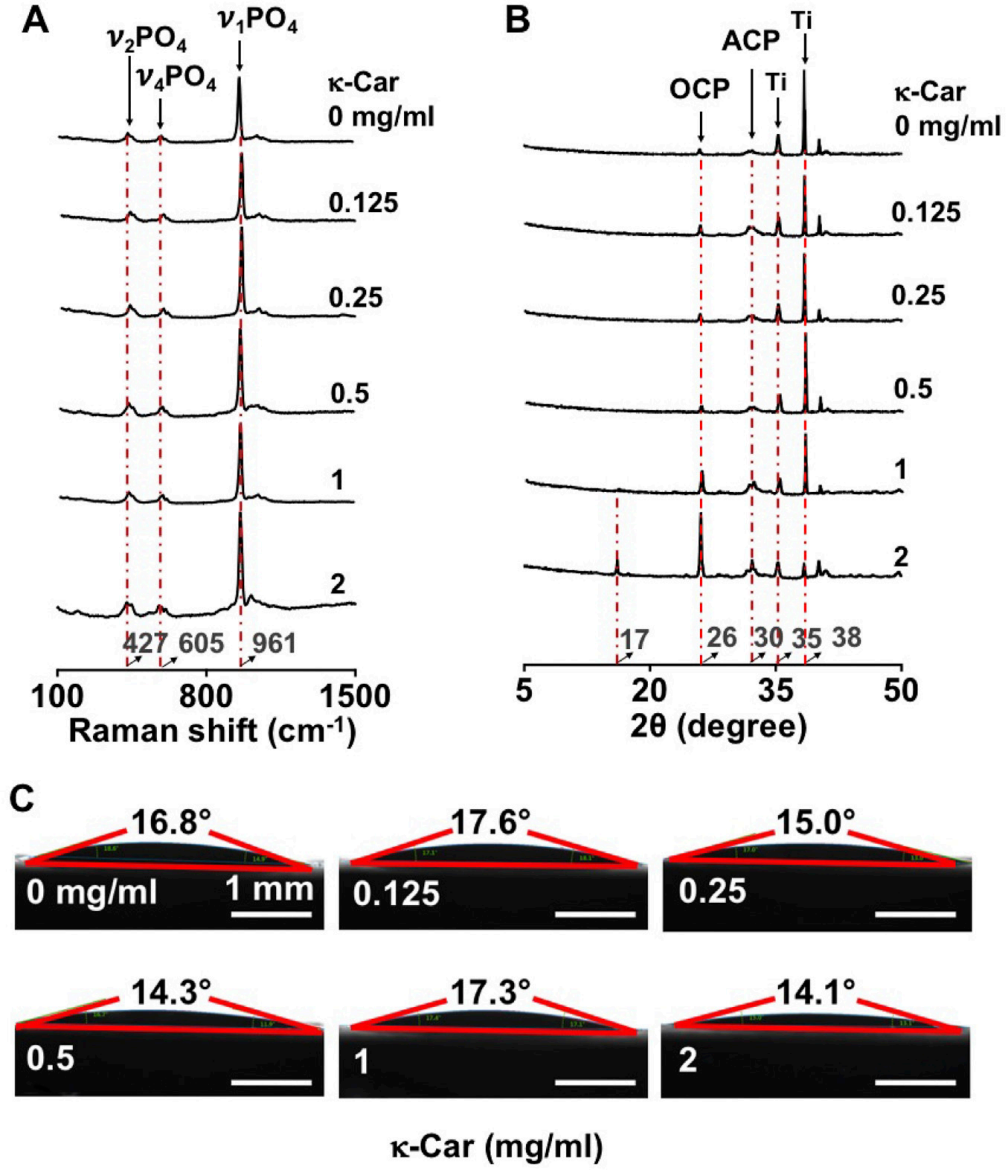
Effect of κ-carrageenan in OCP-coating of titanium discs on the crystalline phase and surface hydrophilicity of OCP-coating. **(A)** Raman spectra. **(B)** XRD spectra. **(C)** Surface hydrophilicity. κ-Car, κ-carrageenan. Bar: 1 mm.

Raman spectroscopy of OCP-coated titanium discs without/with κ-carrageenan was performed to show the chemical composition of OCP, whereby the internal phosphate (PO_4_^3−^) modes were taken into consideration (Akiva et al., 2016). A strong band at 961 cm^−1^ was assigned to the totally symmetric stretching mode (ν1) of PO_4_^3−^ group in OCP. Double (ν2) and triple (ν4) degenerate bending modes were observed at 427 cm^−1^ and 605 cm^−1^. The XRD pattern of OCP-coated titanium discs without/with κ-carrageenan was also evaluated (Figure 2B). The titanium surface pattern showed a bump at 30° typical of a CaP amorphous state. The sharp peak at 26° was typical of triclinic OCP crystal. κ-Carrageenan (0.125–2 mg/ml) increased the intensity of crystallinity peaks corresponding to the crystal structure of OCP, with κ-carrageenan at 2 mg/ml showing the highest peak intensity. OCP containing κ-carrageenan at 2 mg/ml, but not at the other concentrations tested, resulted in a peak at 17° corresponding to diffraction in the OCP structure. The peaks at 35° and 38° were assigned to titanium. Water contact angle measurement revealed that the hydrophilicity of OCP-coated titanium discs without/with κ-carrageenan was similar (Figure 4C).

### 3.3 Protein release

**FIGURE 3:**
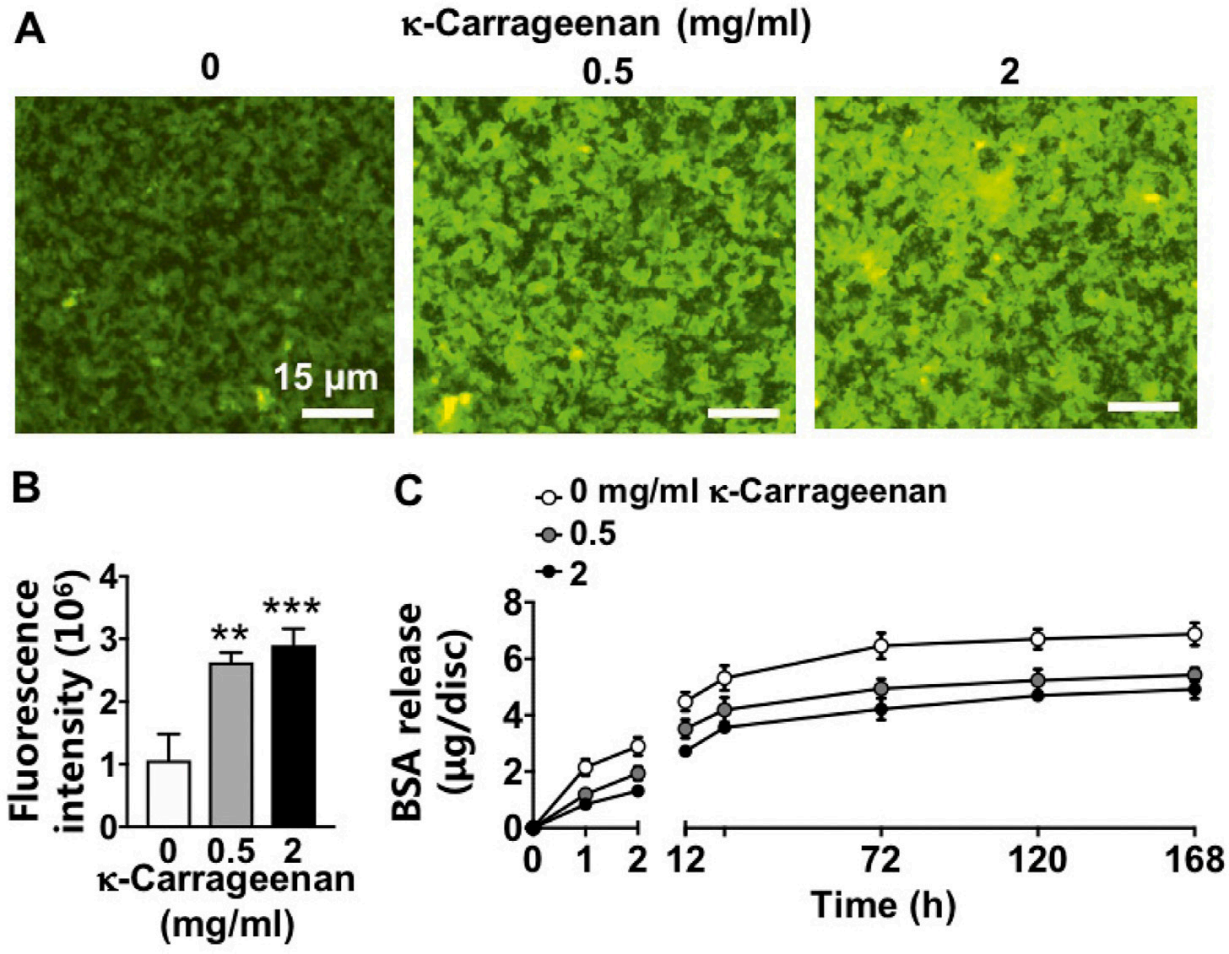
Effect of increasing κ-carrageenan concentration in OCP-coating of titanium discs on BSA release from OCP-coating up to day 7. Total amount of initial BSA incorporated per disc: 10 µg. **(A)** Fluorescent images of FITC-BSA on the surface of OCP-coating. **(B)** Quantification of fluorescence intensity on the surface of OCP-coating. **(C)** BSA release profile. Values are mean ± SD. *n* = 3 from 3 independent experiments. BSA, bovine serum albumin. Significant effect of κ-carrageenan, **p < 0.01, ***p < 0.001. Bar: 15 µm.

FITC-BSA was incorporated in the OCP coating without/with κ-carrageenan, and BSA release kinetics were determined during 7 days (Figure 3). OCP coatings without κ-carrageenan showed less FITC-BSA (top view) compared to OCP coatings with κ-carrageenan at 0.5 mg/ml and 2 mg/ml (most FITC-BSA) after 7 days incubation (Figure 3A). Quantification of the fluorescence intensity at day 7 (Figure 3B) confirmed this observation indicating that BSA release decreased by adding κ-carrageenan to the OCP coating. The BSA release kinetics showed that the inhibition of BSA release by κ-carrageenan occurred especially during the first 2 h of incubation, with the strongest inhibition at 2 mg/ml (Figure 3C).

### 3.4 Pre-osteoblast spreading

**FIGURE 4:**
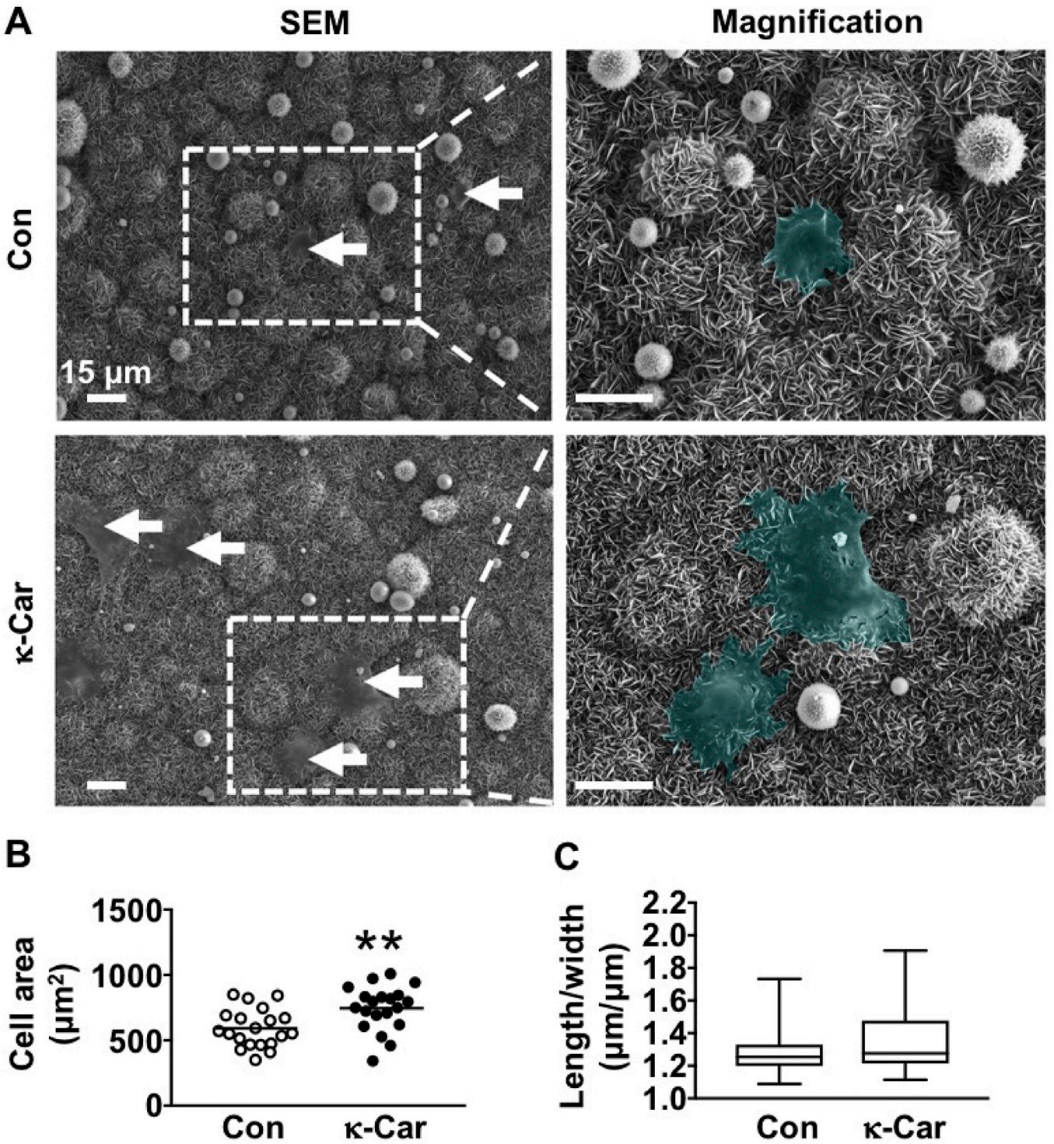
Effect of 2 mg/ml κ-carrageenan in OCP-coating of titanium discs on the spreading of MC3T3-E1 pre-osteoblasts at 1 h **(A)** SEM images of MC3T3-E1 pre-osteoblasts on OCP-coating. Arrows: cells (green). **(B)** Quantification of cell surface area. **(C)** Cell length/width ratio. Values are mean ± SD. *n* = 9 from 3 independent experiments. Significant effect of κ-carrageenan, **p < 0.01. Con, control; κ-Car, κ-carrageenan; SEM, scanning electron microscopy. Bar: 15 μm. Magnification: 4×.

SEM images revealed visual differences between MC3T3-E1 pre-osteoblasts spreading at OCP coating without and with κ-carrageenan (2 mg/ml) at 1 h after seeding (Figure 4A). κ-Carrageenan (2 mg/ml) significantly enhanced the cell surface area by 1.26-fold (Figure 4B). The cell length/width ratio remained unchanged by κ-carrageenan (Figure 4C).

### 3.5 Paxillin protein expression

**FIGURE 5:**
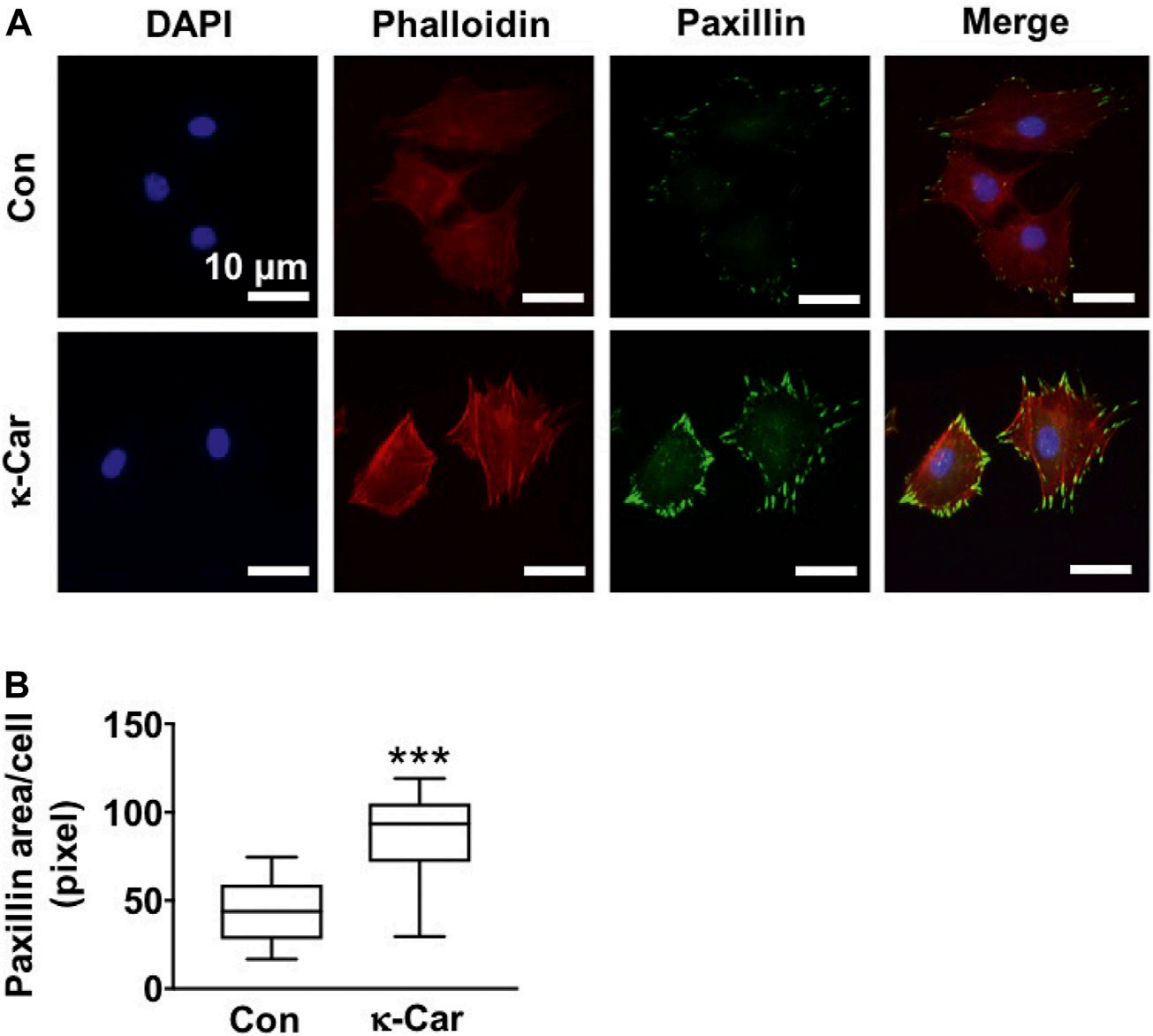
Effect of 2 mg/ml κ-carrageenan in OCP-coating of titanium discs on paxillin expression and distribution of MC3T3-E1 pre-osteoblasts at 1 h. **(A)** Cells stained for DAPI/phalloidin (blue/red) and paxillin (green). **(B)** Quantification of paxillin area in untreated control and κ-carrageenan-treated cells. Values are mean ± SD. *n* = 9 from 3 independent experiments. Significant effect of κ-carrageenan, ***p < 0.001. Con, control; κ-Car, κ-carrageenan. Bar: 10 μm.

Immunofluorescence staining of paxillin revealed clear clusters at the cell boundary of untreated control MC3T3-E1 pre-osteoblasts on OCP-coated titanium discs without/with κ-carrageenan (Figure 5). κ-Carrageenan (2 mg/ml) in the OCP coating resulted in more paxillin dots, resembling short rods (Figure 5A). Quantification of the area covered by paxillin dots indicated a significant (2.03-fold) increase in fluorescent paxillin area per cell after 1 h incubation (Figure 5B).

### 3.6 MC3T3-E1 pre-osteoblast proliferation and metabolic activity

**FIGURE 6:**
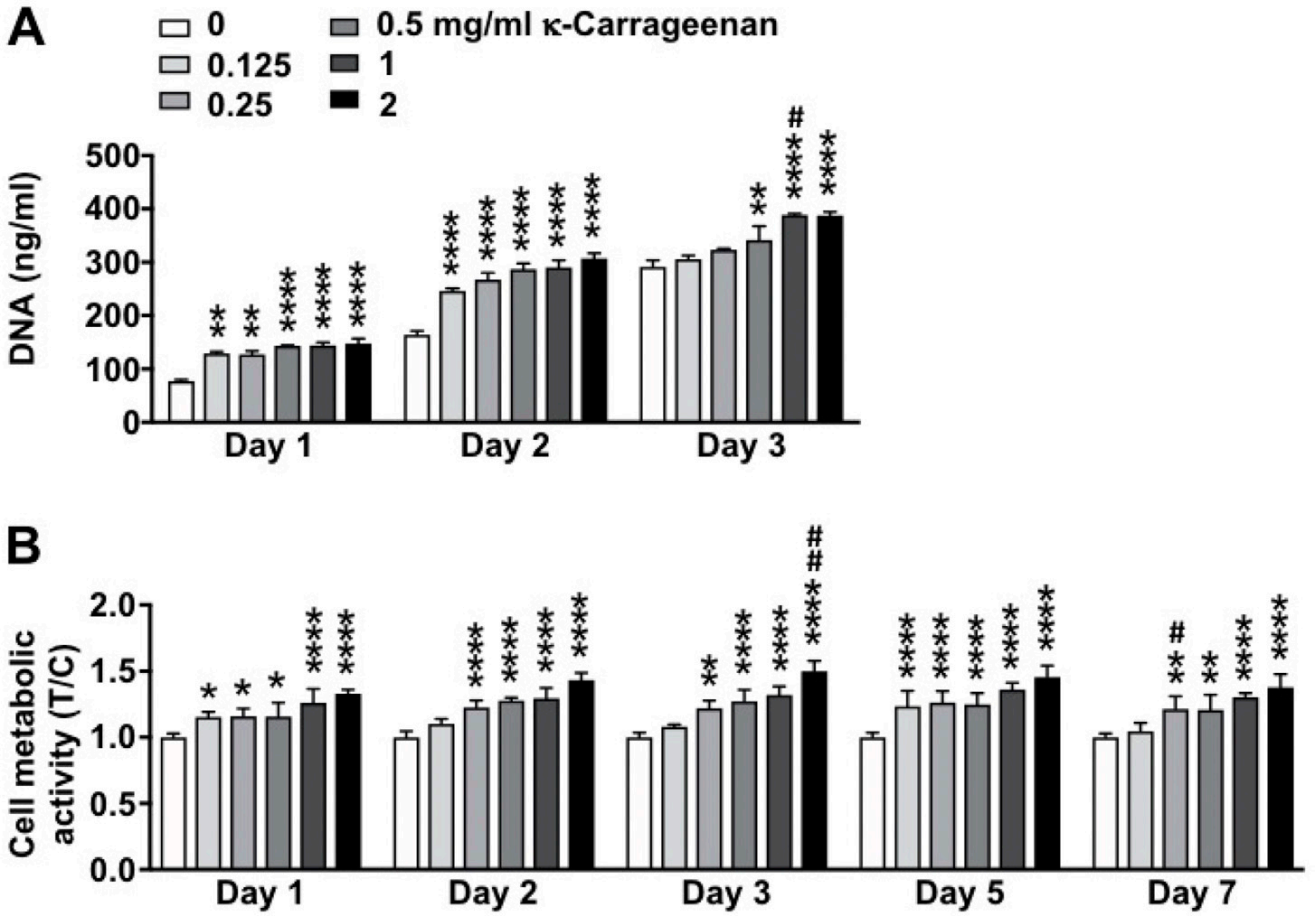
Dose-dependent effect of κ-carrageenan in OCP-coating of titanium discs on MC3T3-E1 pre-osteoblasts proliferation (day 1, 2, 3) and metabolic activity (day 1–7). **(A)** Total cellular DNA content. **(B)** Presto-blue cell metabolic activity. Data are expressed as κ-carrageenan-treated-over-control (T/C) ratio. Values are mean ± SD. *n* = 3 from 3 independent experiments. Significant effect of κ-carrageenan, *p < 0.05, **p < 0.01, ****p < 0.0001, ^#^ *p* < 0.05, ^##^ *p* < 0.01.

The effect of increasing concentrations of κ-carrageenan in OCP coating on MC3T3-E1 pre-osteoblast proliferation and metabolic activity for 3 and 7 days, respectively, was determined (Figure 6). Cell proliferation as assessed by DNA content was most significantly enhanced by treatment with κ-carrageenan at 2 mg/ml from day 1–3 (maximum increase 1.92-fold, day 1) (Figure 6A). MC3T3-E1 pre-osteoblast metabolic activity was most significantly enhanced after treatment with 2 mg/ml κ-carrageenan from day 1–7 (maximum 1.50-fold increase, day 3) (Figure 6B).

### 3.7 Alkaline phosphatase activity and protein staining

**FIGURE 7:**
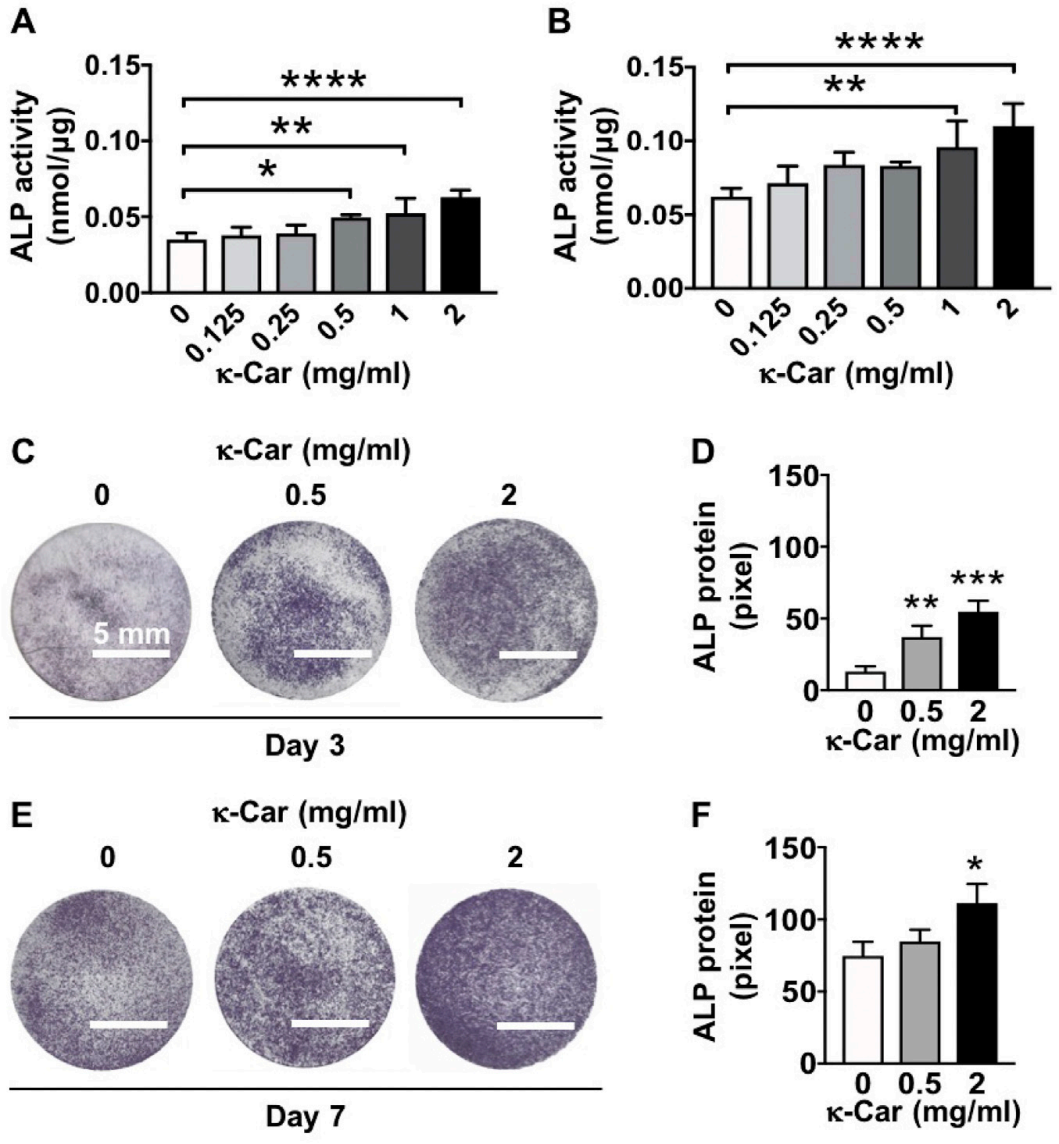
Dose-dependent effect of κ-carrageenan in OCP-coating of titanium discs on ALP activity of MC3T3-E1 pre-osteoblasts at day 3 and 7. **(A)** ALP activity at day 3. **(B)** ALP activity at day 7. **(C)** ALP protein staining at day 3. **(D)** Quantitive colorimetric results of ALP staining at day 3. **(E)** ALP protein staining at day 7. **(F)** Quantitive colorimetric results of ALP staining at day 7. Values are mean ± SD. *n* = 3 from 3 independent experiments. Significant effect of κ-carrageenan, *p < 0.05, **p < 0.01, ***p < 0.001, ****p < 0.0001. ALP, alkaline phosphatase; κ-Car, κ-carrageenan. Bar: 5 mm.

ALP activity in MC3T3-E1 pre-osteoblasts was enhanced by κ-carrageenan at day 3 (0.5, 1, and 2 mg/ml) and day 7 (1 and 2 mg/ml), with 2 mg/ml showing the strongest effect (Figures 7A,B). At day 3, κ-carrageenan (0.5 and 2 mg/ml) also increased ALP protein by 2.85-fold (0.5 mg/ml) and 4.21-fold (2 mg/ml) (Figures 7C,D). At day 7, κ-carrageenan (2 mg/ml) increased ALP protein by 1.49-fold (Figures 7E,F).

### 3.8 Mineralized extracellular matrix production

**FIGURE 8:**
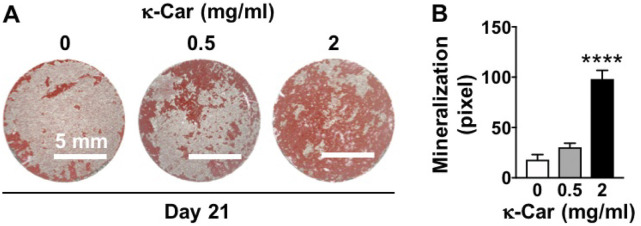
Dose-dependent effect of κ-carrageenan in OCP-coating of titanium discs on extracellular matrix mineralization in MC3T3-E1 pre-osteoblasts at day 21. **(A)** Alizarin red-stained mineralized matrix. **(B)** Quantitive colorimetric results of extracellular matrix mineralization. Values are mean ± SD. *n* = 3 from 3 independent experiments. Significant effect of κ-carrageenan, ****p < 0.0001. κ-Car, κ-carrageenan. Bar: 5 mm.

OCP coating with κ-carrageenan (2 mg/ml) increased matrix mineralization in pre-osteoblasts after 21 days of culture (Figure 8). Matrix mineralization (red) was increased by 5.45-fold, *i.e.* more intense red staining, on OCP-coated titanium discs with κ-carrageenan (Figures 8A,B).

### 3.9 Osteogenic gene expression

**FIGURE 9:**
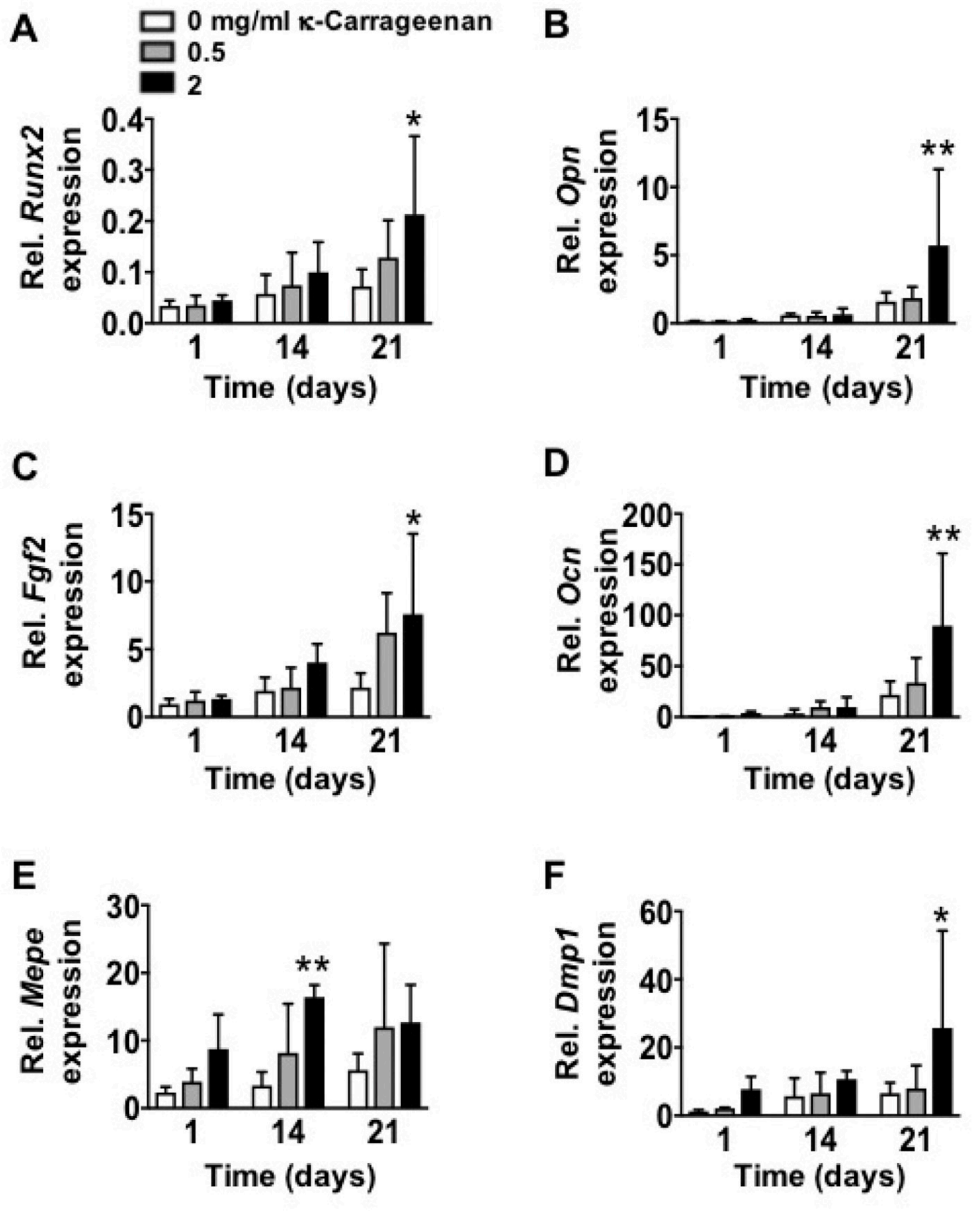
Effect of increasing κ-carrageenan concentration in OCP-coating of titanium discs on expression of osteogenic markers in MC3T3-E1 pre-osteoblasts at day 1, 14, and 21. Expression pattern of **(A)** *Runx2,* **(B)** *Opn,* **(C)** *Fgf2,* **(D)** *Ocn,* **(E)** *Mepe,* and **(F)** *Dmp1*. Values are mean ± SD. *n* = 3 from 3 independent experiments. Significant effect of κ-carrageenan, *p < 0.05, **p < 0.01. *Runx2*, Runt-related transcription factor-2; *Opn*, osteopontin; *Fgf2*, fibroblast growth factor-2; *Ocn*, osteocalcin; *Dmp1*, dentin matrix protein-1; *Mepe*, matrix extracellular phosphoprotein.

Gene expression of all osteogenic markers (*Runx2*, *Opn*, *Fgf2*, *Ocn*, *Mepe*, and *Dmp1*) was similar on OCP-coated titanium discs without/with κ-carrageenan at day 1. Gene expression of all markers was also similar at day 14, except for *Mepe* expression, which was increased (4.93-fold) in pre-osteoblasts on OCP coating with 2 mg/ml κ-carrageenan (Figure 9). OCP coating with κ-carrageenan (2 mg/ml) Increased mRNA levels of *Runx2* (2.94-fold), *Opn* (3.59-fold), *Fgf2* (3.47-fold), *OCn* (3.88-fold), and *Dmp1* (4.59-fold) compared to OCP-coated titanium discs without κ-carrageenan at day 21.

## 4 Discussion

This study aimed to explore whether κ-carrageenan can functionalize OCP coating on a titanium surface to enhance osseointegration of implants. We characterized κ-carrageenan-functionalized biomimetic OCP-coated titanium and tested the effect of carrageenan-functionalization on MC3T3-E1 pre-osteoblast behavior and osteogenic differentiation. We showed, for the first time, that κ-carrageenan can be co-precipitated with an OCP coating on the surface of titanium discs. κ-Carrageenan at all concentrations tested did affect the morphology of the OCP-coated titanium discs, i.e. it changed the more straight sheet-like structure of CaP into a more curved sheet-like structure. κ-Carrageenan significantly reduced the pore size in the sheet-like CaP structure. κ-Carrageenan in the OCP coating did not affect the coating’s Ca/P ratio, chemical composition, or hydrophilicity. Moreover, we found that κ-carrageenan in the OCP coating increased pre-osteoblast spreading, proliferation, and metabolic activity, as well as ALP activity, matrix mineralization, and osteogenic gene expression. Thus, κ**-**carrageenan-functionalization of OCP-coated titanium discs enhanced pre-osteoblast behavior and osteogenic differentiation, suggesting that κ-carrageenan-functionalized OCP coating might improve osseointegration of titanium implants.

In the current study, we functionalized the OCP coating on the surface of titanium discs by coprecipitation with κ-carrageenan. CaP coatings on titanium implant surfaces have been widely investigated to integrate metallic implants with living bone (Das et al., 2019; Pazarceviren et al., 2021). OCP can be deposited onto a substratum using layer-by-layer deposition technology in a simulated *in vivo* microenvironment at 37°C (Wu et al., 2010), rather than at a high temperature (>1,000°C) as needed for other types of CaP coating, e.g. hydroxyapatite or β-tricalcium phosphate (Takahashi et al., 2008). In our study, Raman spectroscopy showed three typical peaks at 427 (cm^−^), 605 (cm^−^), and 961 (cm^−^) of phosphate (PO_4_^3−^) modes, the typical chemical composition of OCP. XRD showed a typical peak at 30° of amorphous CaP, as well as a typical peak at 26° of the crystal phase of OCP. These results indicate successful incorporation of κ-carrageenan in the OCP coating on a titanium surface using a biomimetic co-precipitation technique.

We found that κ-carrageenan at all concentrations tested did affect the morphology of OCP-coated titanium discs by changing the more straight sheet-like structure of CaP into a more curved sheet-like structure. Material surface morphology affects cell-material interaction (Zamani et al., 2018), as well as the response of bone tissue towards titanium implants *in vivo* (Levin et al., 2022). Moreover, κ-Carrageenan significantly reduced the pore size in the CaP sheet-like network structure, suggesting increased density of the OCP sheets, which might facilitate pre-osteoblast adhesion and spreading onto the OCP coating. Adhesion and spreading of osteoprogenitors are affected by integrin binding and focal adhesion formation (Kim et al., 2012). Paxillin, a fundamental focal adhesion-associated adapter that connects structural and signaling components, plays a major role in integrating multiple signals from the microenvironment. In the current study, the more curved OCP sheet-like structure was associated with the presence of κ-carrageenan in the coating, as well as with enhanced pre-osteoblast spreading and paxillin expression. This might indicate that κ-carrageenan in the OCP coating provides more sites for osteoblast recruitment, which might lead to enhanced bone formation, which would be beneficial for osseointegration.

We found that κ-carrageenan did not affect the Ca/P ratio, chemical composition, or hydrophilicity of the OCP coating. The molar Ca/P ratio of CaP deposited onto titanium has been reported to vary between 1.31 and 2.05, being in the range of apatitic CaP (Nourmohammadi et al., 2017). We found an OCP Ca/P molar ratio of 1.40 ± 0.06 (mean ± SD, *n* = 3). This ratio resembles the reported OCP molar CaP ratio of 1.33 (Nourmohammadi et al., 2017), indicating that κ-carrageenan did not affect the chemical composition of the OCP coating, and thus can be successfully incorporated in the OCP coating. Hydrophilic surfaces tend to enhance cell adhesion, proliferation, differentiation, and bone mineralization in the early stage compared to hydrophobic surfaces (Arya & Kumar, 2020). We showed that κ-carrageenan did not affect the hydrophilicity of the coating, i.e. κ-carrageenan-functionalized OCP coating is attractive for osteoblast adhesion, and might be advantageous for osseointegration.

We found that the addition of κ-carrageenan to the OCP coating slowed down protein (BSA) release during the first 2 h of incubation, with the strongest inhibition at 2 mg/ml. This indicates that the protein release characteristics by addition of κ-carrageenan are beneficial for bone tissue engineering purposes. Biomimetic CaP-based coatings on implants can serve as a vehicle for the targeted delivery of osteogenic agents, e.g., BMP2, to the local implant environment (Liu et al., 2018). BMP2 stimulates both osteoprogenitor and osteoclast recruitment, proliferation, and differentiation. Thus, high concentrations of BMP2 released might promote the resorption of newly formed bone almost as soon as it has been laid down. Moreover, the high cost and local/systemic adverse effects of BMP2 limit its clinical application. Currently, natural polymers are widely used in the food industry, chemical engineering, and pharmaceutical applications (Caillol, 2020). These natural polymers are also used as bioactive interphases to enhance rapid, robust, and functional osseointegration between bone and implants (Caillol, 2020). One of these natural polymers is κ-carrageenan, which is naturally available in high amounts, and easy to extract (Du et al., 2016). Therefore, κ-carrageenan might be a promising factor for cost-effective clinical application. We found that κ-carrageenan slowed down the release of protein (BSA) during the early stage of release from the OCP coating. This indicates that our new OCP coating containing κ-carrageenan may be able to control the precise release rate of other osteogenic agents, e.g., BMP2, or drugs that are important for bone regeneration.

Cell adhesion, spreading, and metabolic activity are directly correlated with osseointegration and implant success (Zamani et al., 2018). Our data showed that κ-carrageenan-functionalized OCP coating on titanium discs robustly promoted pre-osteoblast spreading, as well as increased pre-osteoblast proliferation and metabolic activity, suggesting that κ-carrageenan-functionalized OCP coating is biocompatible and attractive to preosteoblasts and therefore likely to result in osseointegration and implant success. Moreover, biomimetic co-precipitation-based κ-carrageenan functionalization of OCP might also improve precursor cell spreading as well as metabolic activity, which would provide a basis for further research on the suitability of κ-carrageenan functionalization of OCP coating on dental titanium implants.

Osteogenic differentiation of attached and proliferated precursor cells into osteoblasts depositing new bone on the implant surface is another key biological process required for implant osseointegration (Zamani et al., 2018). We found that κ-carrageenan-functionalized OCP coating on titanium discs induced osteogenic differentiation of pre-osteoblasts, as indicated by the enhanced ALP activity, matrix mineralization, and osteogenic gene expression. κ-Carrageenan in the OCP coating enhanced osteogenic gene expression (*Mepe, Runx2*, *Opn*, *Fgf2*, *Ocn*, and *Dmp1)* in the long-term (14–21 days). *Runx2* is a key transcription factor associated with bone-related cell differentiation. In the cell cycle, *Runx2* plays a vital cell proliferation regulatory role in osteoblasts (Lucero et al., 2013). We found that κ-carrageenan in the OCP coating increased *Runx2* mRNA expression, which might be due to the effect of ascorbic acid and β-glycerophosphate in the osteogenic medium taken up by pre-osteoblasts after seeding on OCP coating containing κ-carrageenan at 21 days. *Opn* has an crucial role in endocrine-regulated and neuron-mediated bone mass, but is also involved in adhesion, proliferation, and migration of different bone cells (Vancea et al., 2021). *Ocn* has a significant role in the regulation of bone metabolism, and is secreted only by osteoblasts (Lee et al., 2007). *Fgf2* affects gene expression in osteoblasts in a biphasic fashion, depending on the osteoblast maturation stage (Pitaru et al., 1993). *Ocn* is involved in calcium ion homeostasis and bone mineralization, as is *Fgf2* (Globus et al., 1989). *Dmp1* is a non-collagenous extracellular matrix mineralization protein found in dentin and bone, similar as bone sialoprotein and *Opn*, which combines ligand N-linked glycoprotein family, and is part of the small integrin-binding ligand (Fisher et al., 2004). These osteogenic genes (*Opn, Ocn, Fgf2, and Dmp1*) are well related to extracellular matrix mineralization in the long term. We found that κ-carrageenan enhanced expression of these osteogenic genes at 21 days. *Mepe* has a multifunctional role in the regulation of cell signaling, mineral homeostasis, and mineralization (Chrepa et al., 2017). In this study, κ-carrageenan did affect *Mepe* gene expression at 14 days, but not at 21 days, suggesting that κ-carrageenan affects *Mepe* expression only in the short term. *Mepe* belongs to the small integrin-binding ligands and influences the adhesion and differentiation of cells from the osteoblast cell lineage leading to increased bone formation (Sprowson et al., 2008). Therefore, the increased *Mepe* expression might be related to the improved cell spreading on the OCP coating containing κ-carrageenan. ALP activity and matrix mineralization are important for bone regeneration (Moghaddaszadeh et al., 2021). We found that κ-carrageenan in the OCP coating enhanced ALP activity and ALP protein in the short term (3, 7 days), as well as enhanced matrix mineralization in the long term (21 days) in pre-osteoblasts, suggesting stimulation of bone formation. κ-Carrageenan has already been shown to have chondrogenic, soft tissue, and osteogenic regenerative potential (Nourmohammadi et al., 2017; Gonzalez Ocampo et al., 2019; Mokhtari et al., 2019). κ-Carrageenan with gelatin, and sericin hydrogel composites improve cell viability of cryopreserved Saos-2 cells (Ashe et al., 2020). Moreover, we have shown earlier that exogenously added κ-carrageenan promotes migration, adhesion, spreading, and osteogenic differentiation of MC3T3-E1 preosteoblasts *in vitro* (Cao et al., 2021). These findings indicate that κ-carrageenan-functionalization of OCP-coated titanium surface results in implant osteoinductivity, thereby improving osseointegration.

In this study, we successfully incorporated κ-carrageenan in the OCP coating on a titanium surface using a biomimetic co-precipitation technique. We found that κ-carrageenan in the OCP coating increased pre-osteoblast spreading, proliferation, and metabolic activity, as well as ALP activity, matrix mineralization, and osteogenic gene expression suggesting that κ-carrageenan-functionalized OCP coating might improve osseointegration of titanium dental or orthopaedic implants. We found a positive effect of κ-carrageenan in OCP-coating on most pre-osteoblast activities. This is likely due to the fact that κ-carrageenan contains abundant sulfate groups, which mimic the charged proteins present in the extracellular matrix and ensures the practicality of κ-carrageenan for bone tissue engineering. This fits earlier literature findings that the structural similarity between κ-carrageenan and glycosaminoglycans improves osteoblast adhesion and proliferation (Gonzalez Ocampo et al., 2019). However, the exact molecular mechanisms for bone regeneration need further investigation. An ideal coating of a medical implant is biocompatible, osteoconductive, osteoinductive, and has optimal stiffness. During implantation, the surgical procedure may destroy the surface topography of the coating. Future studies will test the stiffness, thickness, and stability of the coating, and address the underlying mechanism of κ-carrageenan-increased MC3T3-E1 cell spreading, proliferation, and osteogenic differentiation. Recent advances in the field of cell-based therapeutics provide new perspectives for oral tissue regeneration. The development of large animal models, which overcome the limitations of rodent models and allow to emulate clinical situations, is crucial for the validation of regenerative strategies to move toward clinical application (Mangione et al., 2022). Canine models are successfully used in all oral tissue regeneration, notably implantology studies (Uijlenbroek et al., 2022). The findings of this study will be further verified using a canine mandibular peri-implant bone defect healing model.

## 5 Conclusion

In this study, we successfully incorporated κ-carrageenan in the OCP coating on a titanium surface using a biomimetic co-precipitation technique. κ-Carrageenan in OCP coating on titanium discs modified the morphology and microstructure of the OCP-coating, and enhanced pre-osteoblast metabolic activity, proliferation, and osteogenic differentiation. These findings suggest that κ-carrageenan-functionalized OCP coating may be promising, especially in the short term, for *in vivo* improvement of titanium dental and orthopaedic implant osseointegration.

## Data Availability

The original contributions presented in the study are included in the article/Supplementary Material, further inquiries can be directed to the corresponding authors.
