# Supplementary material for: Kappa-carrageenan-Functionalization of octacalcium phosphate-coated titanium Discs enhances pre-osteoblast behavior and osteogenic differentiation
Source: Front Bioeng Biotechnol. 2022 Oct 20;10:1011853. doi: 10.3389/fbioe.2022.1011853 (PMC9632979; doi:10.3389/fbioe.2022.1011853)

Supplementary Figure 2. Effect of κ-carrageenan at an increasing concentration in a super-saturated OCP solution on the solubility of OCP. Top view images show beakers containing OCP solution with κ-carrageenan at 0.5-5 mg/ml after 48 h of incubation in the presence of titanium discs. CaP, calcium phosphate.

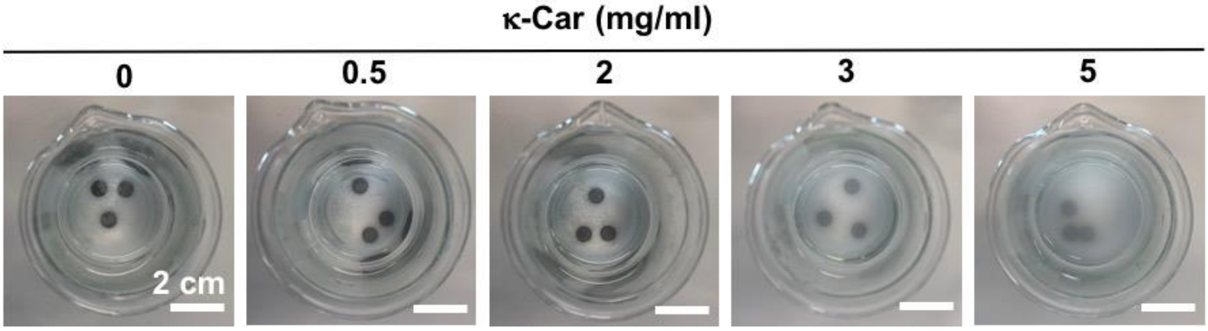

Supplement: Supplementary file 1 [file DataSheet2.PDF]
