# Supplementary material for: Kappa-carrageenan-Functionalization of octacalcium phosphate-coated titanium Discs enhances pre-osteoblast behavior and osteogenic differentiation
Source: Front Bioeng Biotechnol. 2022 Oct 20;10:1011853. doi: 10.3389/fbioe.2022.1011853 (PMC9632979; doi:10.3389/fbioe.2022.1011853)

Supplementary Figure 3. Effect of κ-carrageenan in OCP coating on protein adsorption onto titanium discs after 1, 12, or 24 h incubation in α-MEM with 10% FCS. Values are mean±SD. n=3 from 3 independent experiments. Significant effect of k-carrageenan, ##p<0.01, ****p<0.0001. FCS, fetal calf serum.

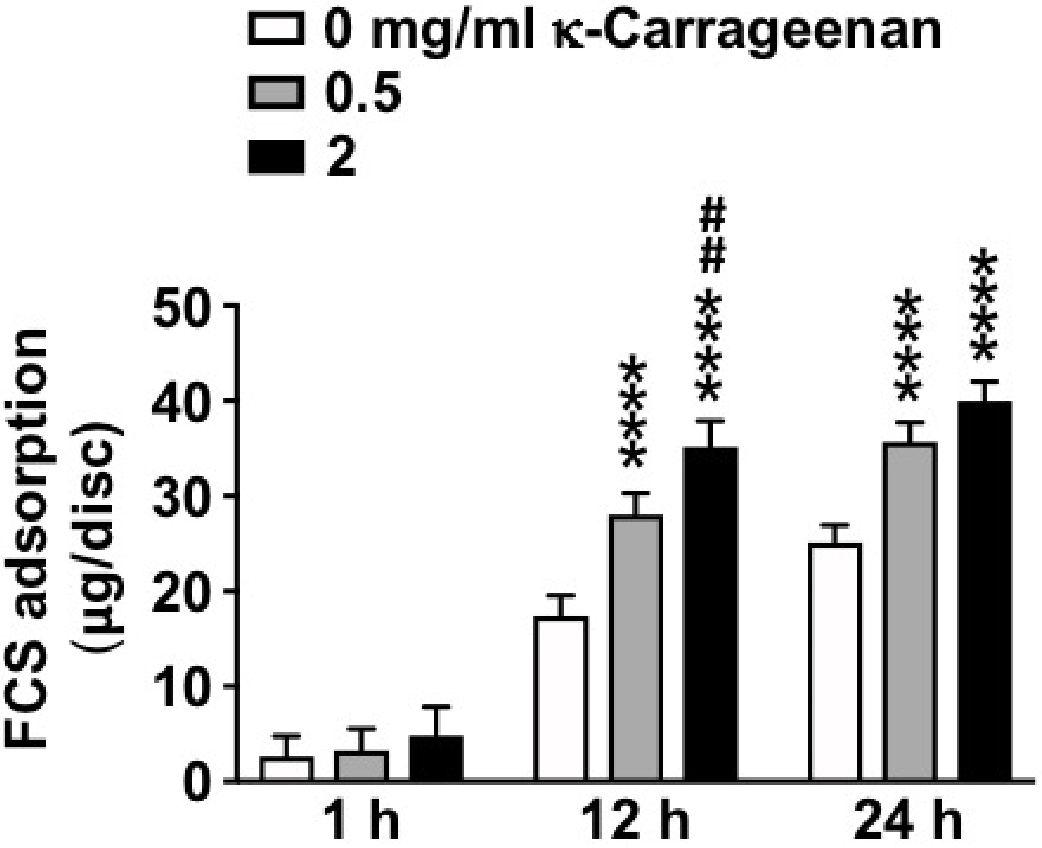

Supplement: Supplementary file 2 [file DataSheet3.PDF]
