# Supplementary figures and images for: Kappa-carrageenan-Functionalization of octacalcium phosphate-coated titanium Discs enhances pre-osteoblast behavior and osteogenic differentiation
Source: Front Bioeng Biotechnol. 2022 Oct 20;10:1011853. doi: 10.3389/fbioe.2022.1011853 (PMC9632979; doi:10.3389/fbioe.2022.1011853)

**Supplementary Figure 1. Graphical abstract**

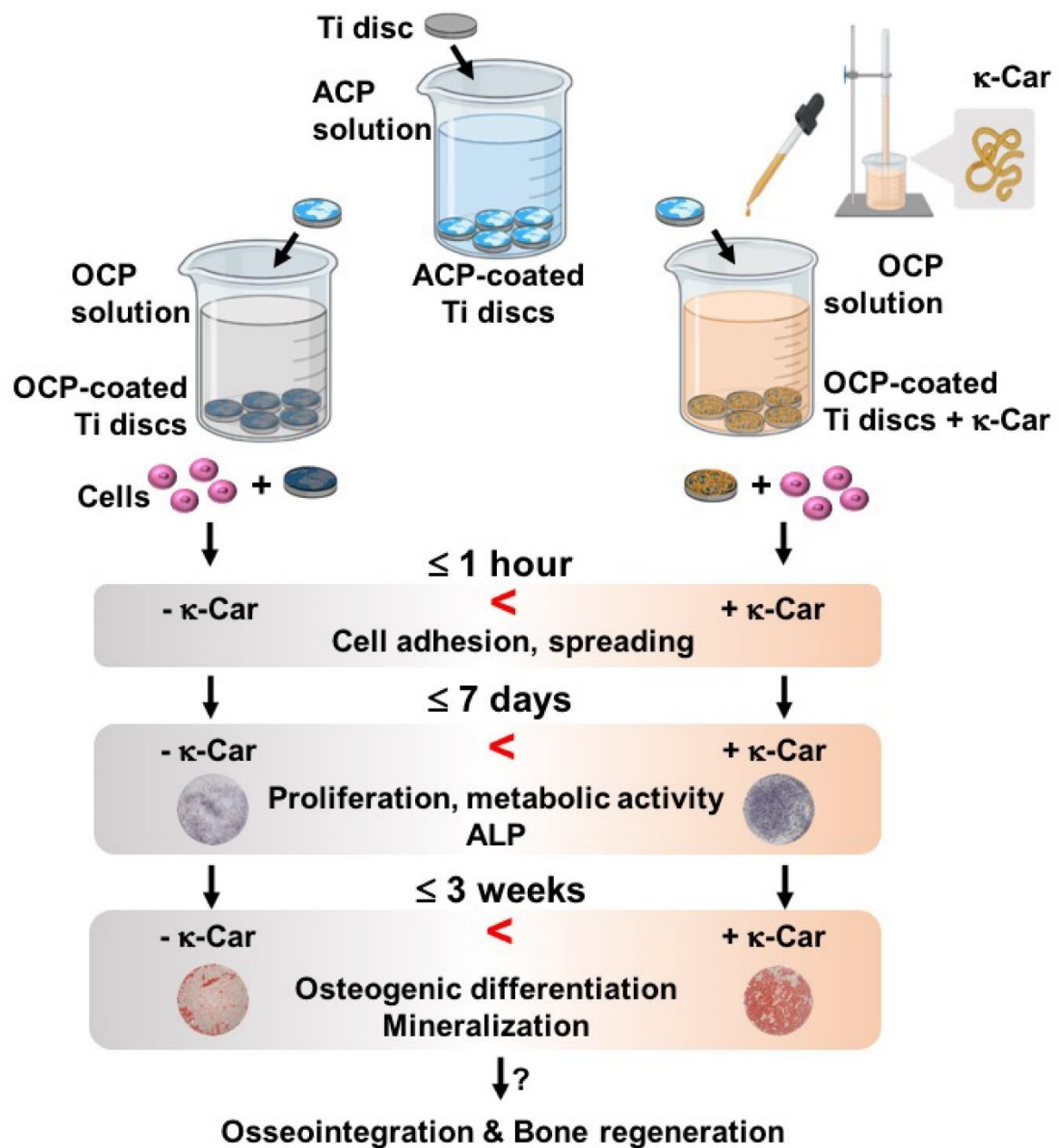

Supplement: Supplementary file 3 [file DataSheet1.PDF]
